# Population Prevalence of Trachoma in Nine Rural Non-Indigenous Evaluation Units of Brazil

**DOI:** 10.1080/09286586.2021.1941127

**Published:** 2021-10-29

**Authors:** Célia Landmann Szwarcwald, Maria de Fátima Costa Lopes, Paulo Roberto Borges de Souza Junior, Daniela Vaz Ferreira Gómez, Expedito José de Albuquerque Luna, Wanessa da Silva de Almeida, Giseli Nogueira Damacena, Joana da Felidade Ribeiro Favacho, Paulo Germano de Frias, Robert Butcher, Sarah Boyd, Ana Bakhtiari, Rebecca Willis, Cristina Jimenez, Emma Harding-Esch, Martha Idalí Saboyá-Díaz, Anthony W. Solomon

**Affiliations:** a Institute of Scientific and Technological Communication and Information in Health, Oswaldo Cruz Foundation, Rio de Janeiro, Brazil; b Coordination of Surveillance of Zoonoses and Vector Transmission Diseases, Department of Immunization and Communicable Diseases, Health Surveillance Secretariat, Ministry of Health, Brasília, Brazil; c Department of Preventive Medicine, Medical School, University of São Paulo, São Paulo, Brazil; d Evandro Chagas Institute (IEC), Health Surveillance Secretariat, Ministry of Health, Belém-Pará, Brazil; e Board of Education and Research, Study Group on Health Assessment and Management, Professor Fernando Figueira Integral Medicine Institute (IMIP), Recife, Brazil; f Clinical Research Department, London School of Hygiene & Tropical Medicine, London, UK; g International Trachoma Initiative, Task Force for Global Health, Atlanta, Georgia, USA; h Sightsavers International, Haywards Heath, UK; i Neglected, Tropical, and Vector-Borne Diseases Unit, Communicable Diseases and Environmental Determinants of Health Department, Pan American Health Organization (PAHO), Washington, DC, USA; j Department of Control of Neglected Tropical Diseases, World Health Organization, Geneva, Switzerland

**Keywords:** Trachoma, trichiasis, Brazil, prevalence, neglected tropical diseases, elimination, WASH, Tropical Data

## Abstract

**Purpose:**

To assess the contemporary prevalence of trachoma in Brazil’s non-indigenous population, surveys of those thought to be at greatest risk of disease were conducted.

**Methods:**

Rural census tracts of non-indigenous population from nine mesoregions were selected to compose the survey evaluation units (EUs) by considering previously endemic municipalities at greatest risk of trachoma. In each of the nine EUs, we conducted a population-based prevalence survey. Every resident of selected households aged ≥1 year was examined for trachomatous inflammation — follicular (TF) and trachomatous trichiasis (TT). Additionally, data were collected on household-level access to water, sanitation, hygiene (WASH) and education.

**Results:**

A total of 27,962 individuals were examined across nine EUs. The age-adjusted TF prevalence in 1–9-year-olds was <5% in each EU. The age- and gender-adjusted prevalence of TT unknown to the health system in ≥15-year-olds was <0.2% in eight EUs; in one EU, it was 0.22%. The median number of households surveyed per EU with access to an improved drinking water source within a 30-minute roundtrip of the house was 66%. School attendance was >99% of surveyed children.

**Conclusions:**

The prevalence of TF was well below the target for elimination as a public health problem in all EUs. Because EUs surveyed were selected to represent the highest-risk non-indigenous areas of the country, TF prevalence is unlikely to be ≥5% in non-indigenous populations elsewhere. In one EU, the prevalence of TT was above the target threshold for elimination. Further investigation and possibly improvement in TT surgical provision are required in that EU.

## Introduction

Trachoma is a chronic inflammatory disease of the eye caused by the intracellular bacterium *Chlamydia trachomatis*. Active (inflammatory) trachoma is characterized by the formation of conjunctival follicles, which resolve spontaneously after infection is cleared. Ocular *C. trachomatis* is thought to be primarily transmitted from person to person by transfer of ocular and nasal secretions during direct contact between individuals, via eye-seeking flies or in fomites, such as face cloths or pillow cases,^1–4^ As such, trachoma disproportionately affects poor communities where resources limit personal hygiene and sanitation.^5–7^ Repeated infections lead to conjunctival scarring which can, in severe cases, cause entropion (where the eyelid margin folds in towards the eye) and trichiasis (where the eyelashes touch the eyeball). Eyelash abrasion damages the cornea, which reduces visual acuity and may lead to blindness.^8^ Many rounds of reinfection are thought to be needed to develop significant conjunctival scarring,^9^so trachoma poses a threat to sight only when ocular *C. trachomatis* occurs commonly within children; isolated cases of trachoma outside endemic areas are uncommon.^10^

The likely public health impact of trachoma is commonly assessed using the World Health Organization (WHO) simplified grading system. This is a set of five signs which covers the early (trachomatous inflammation — follicular [TF], trachomatous inflammation — intense [TI]) and late (trachomatous scarring [TS], trachomatous trichiasis [TT], corneal opacity [CO]) stages of trachoma.^11,12^

In 2010, trachoma was thought to be responsible for visual impairment in 1.4 million people, and irreversible blindness in another 450,000, worldwide,^13^ In March 2019, it was estimated that 142 million people lived in trachoma-endemic areas worldwide,^14^ WHO and partners have targeted trachoma for elimination as a public health problem globally. To declare elimination, countries must meet three criteria: (1) a prevalence of TF among children aged 1–9 years <5% in each formerly-endemic district; (2) a prevalence of TT unknown to the health system in people aged ≥15 years <0.2% in each formerly-endemic district; and (3) a defined strategy to identify and manage incident TT cases.^15^ Collaborations have been developed and best-practice guidance published to support countries to estimate prevalence in suspected-endemic areas.^16–19^

A centralized model of care for the control of trachoma began in Brazil in 1923 and continued until 1998, delivered by the Federal Government through vertical campaigns. Trachoma surveillance and control activities in Brazil have declined since the 1970s, although, according to the prevalence survey conducted from 1974–1976, some high-prevalence states remained, especially in the North region.^20^ Trachoma was considered eliminated in the state of São Paulo in the 1970s, and this was subsequently assumed to be true for the entire country. The disease was neglected, and deprioritised from Ministry of Health surveillance protocols. At the beginning of the 2000s, in order to understand the burden of trachoma among children, the Ministry of Health surveyed a sample of schools in 1,514 municipalities known to have a high proportion of people living in resource-poor settings. The survey of school children included indigenous children who are thought to be at higher risk of trachoma.^21–23^ All cases found in the survey were treated with antibiotics to proactively control disease and reduce transmission,^24^ In seven of 19 (37%) of surveyed states, prevalence of TF among examined school children was ≥5%.^25–27^

These findings gave trachoma visibility in the 2000s, triggering a strengthening of trachoma surveillance and control activities nationally.^28^ Cases found in surveillance activities and/or campaigns started to be reported in the Brazil Information System for Notifiable Diseases (SINAN) and cases were monitored temporally. The current strategy for eliminating trachoma in Brazil consists of active case finding and contact tracing to reduce transmission of infection in endemic areas through antibiotic treatment of individuals with active trachoma and their contacts.^24^

SINAN data from almost 1,000 municipalities that performed trachoma surveillance activities from 2008–16 show a sharp decrease in the percentage of trachoma cases found over that period. However, because trachoma disproportionately affects impoverished communities where public health service access is poor, sampling methodologies that are not population-based, such as school sampling or disease notification by clinical staff, risk introducing bias. Given this limitation, two population-based household surveys were conducted in the states of Pernambuco and Tocantins in 2014 and 2015.^5^ However, these surveys were not designed to current international standards; in particular, graders were not standardised.^17^ For those reasons, and because they were carried out in only two states, further studies with a larger geographical scope and following the methodological standards set by WHO^16^ were deemed to be necessary to support a future claim of elimination of trachoma as a public health problem in Brazil.

The aim of this study was to assess whether disease prevalence targets for trachoma elimination as a public health problem had been met in the non-indigenous Brazilian population in nine areas thought to be at risk of trachoma. Here, we describe the methodology used and the results obtained.

## Materials and methods

### Study design

A cross-sectional observational study was conducted in 2018–9 to estimate the prevalence of TF in children aged 1–9 years and the prevalence of TT in adults aged ≥15 years in supposedly endemic areas. The design of each survey conformed with WHO recommendations, being based on that of the Global Trachoma Mapping Project (GTMP)^16,17,19^ with additional refinements developed by Tropical Data (www.tropicaldata.org).

The project was developed by the Oswaldo Cruz Foundation and the Health Surveillance Secretariat of the Ministry of Health (SVS/MS) and revised and implemented with the support of Tropical Data. The protocol was approved by the Research Ethics Committee of the Oswaldo Cruz Foundation (Declaration No. 2,742,820) and the Pan American Health Organization (2018–06-0045). Tropical Data survey support was approved by the London School of Hygiene & Tropical Medicine Observational Ethics Committee (16105).

### Definition and selection of evaluation units

Brazil is divided by the Brazilian Institute of Geography and Statistics into different geographical units: macroregions, mesoregions and microregions (which have geographical but no political or administrative significance); and states and municipalities (which do have political and/or administrative significance). Municipalities are subdivided into census tracts, which are classified as rural or urban and indigenous or non-indigenous.

Data from prevalence surveys and pro-active search of trachoma cases carried out in some Special Indigenous Health Districts point to high levels of trachoma prevalence in the indigenous population, higher than those of Brazil’s non-indigenous population.^27,28^ However, the demographic characteristics of the indigenous population, the geographical spread of indigenous villages in the Brazilian territory, the specificities related to the organization of health services and to the research approval process, which needs approval by the National Research Ethic Commission (CONEP) and authorization by the National Indigenous Foundation (FUNAI) for all team members to enter indigenous lands, led us to divide the project in two: the first in the non-indigenous population (described herein) and the second in the indigenous population. This second project component will be carried out as a subsequent package of work.

Non-indigneous rural census tracts thought to be at epidemiological or social risk of trachoma were subjected to a preliminary desk evaluation. Epidemiological risk was defined as TF prevalence in school children >10% in the surveys which took place from 2002–2008^27^ or large proportions of children found to have TF in surveillance activities undertaken from 2008–2016. Social risk was defined by poverty and sanitation indicators: (1) mean nominal monthly income of people aged ≥10 years less than one-quarter of the minimum wage; and (2) <30% of households having a piped water supply. For the purposes of the survey, evaluation units (EUs) were defined at the mesoregion level. Eight mesoregions with at least one municipality meeting the criteria for epidemiological risk and with the largest number of non-indigenous census tracts at social risk among all state mesoregions were selected in the following states: Acre, Amazonas, Roraima, Pará, Maranhão, Ceará, Alagoas and Pernambuco. Additionally, to include an EU not previously considered to be trachoma-endemic, one mesoregion was selected in the state of Bahia meeting the criteria for social risk of trachoma but without any municipalities meeting the definition of epidemiological risk.

In each EU, a two-stage sampling strategy was implemented. In the first stage, 30 non-indigenous rural clusters were selected systematically with probability proportional to their resident population size, determined by the 2010 census.^29^ Within each cluster, for the second sampling stage, about 30 households were selected by compact segment sampling. This involved the cluster being subdivided into segments of 30 neighbouring households (or the nearest practical approximation of 30 households) and selection, by simple random sampling, of one segment. All households in the selected segment were invited to participate, leading to inclusion of a total of about 900 households in each EU.

### Data collection

Field work was carried out in three EUs from September–December 2018. Field work in the remaining six EUs took place between August–October 2019. Field teams each consisted of a recorder, a trachoma grader, and a local community health agent, who accompanied the team to facilitate fieldwork at the selected sites. All team members were standardised on the protocol procedures. Trachoma graders and recorders were standardized according to WHO recommendations and Tropical Data manuals.^30^

Additionally, field teams were supervised by two supervisors and two ophthalmologists supported the evaluation and referral of TT cases to health services.

On approaching each selected household, contact was made with the household head and residents, and the study objectives, procedures, and the importance of participation were explained. All residents aged one year or more were invited to participate. Before taking part, each adult resident (≥18 years) was asked to read the Free and Informed Consent Terms and give their written consent to participate. Adults also consented to the participation of residents aged <18 years under their responsibility. For residents aged 7–17 years, the Terms of Assent were presented and signed when they agreed to participate.

After consenting to participate in the research, the household’s GPS coordinates were captured and an interview was conducted with a key informant concerning household availability of water for drinking and washing and type of sanitation and handwash facilites (WASH variables). This questionnaire was translated from the Tropical Data household questionnaire, itself adapted from the WHO/UNICEF Joint Monitoring Programme (JMP) questionnaire, and adapted (with minimal modifications) to suit the Brazilian setting. During the interview, household heads were asked whether they received monthly visits from community health agents.

Demographic information was collected about all residents (both present and absent) aged ≥1 year. Children aged 5–9 years were asked whether they regularly attended school. When one or more residents were absent, one return visit at the end of the day was scheduled.

Data were recorded by the recorder using the Tropical Data smartphone application. Data were encrypted and transmitted to secure Cloud-based servers for cleaning, storage and analysis by the Tropical Data data team in collaboration with the Oswaldo Cruz Foundation and the Ministry of Health. This system enabled real-time fieldwork monitoring and adherence to protocols previously agreed.

### Clinical examination

Clinical grading was conducted by graders who had successfully completed the Tropical Data training programme and achieved an inter-grader agreement (IGA) kappa score of ≥0.7 compared to a certified Tropical Data grader trainer in the grading of 50 school children of whom at least 10% had TF.^30^ School children were pre-screened the week before the IGA to ensure there were sufficient cases of TF, in line with Tropical Data guidelines. External examination of both eyes of all consenting residents aged ≥1 year was undertaken using 2.5× magnifying loupes and ambient or torch illumination. Presence or absence of TT, TF and TI, defined according to the WHO simplified grading system,^11^ was recorded for each examined eye.

Where TF or TI were identified, the grader explained the finding to the individual or their parent/guardian and offered management with azithromycin at no cost to the participant. Children with TF or TI and their household members received antibiotics according to age and weight,^31^ as regulated by the Health Surveillance Secretariat, Ministry of Health.^32^

When TT was identified, the upper tarsal conjunctiva was examined for signs of TS and additional questions were asked to identify whether or not that individual had previously been offered management (i.e., whether they were “unknown to the health system”).^33^

Contact information of TT cases was recorded and sent to local health authorities – the Municipal and State Health Secretariats – responsible for referring them to ophthalmological assessment and to surgery if needed.

The teams were also trained to deliver specific health education actions for the control and prevention of trachoma. These educational actions focused on improving personal hygiene, with an emphasis on encouraging facial cleanliness and other forms of disease prevention.

### Data analysis

The complex sampling design was considered in the statistical analysis. Adjusted prevalence estimates were calculated in the statistical software R using Tropical Data algorithms, which are based on those of the GTMP^17^ and produce EU-level means of adjusted cluster-level proportions. Prevalence of TF in those aged 1–9 years was adjusted for age in one-year bands. Prevalence of TT in those aged ≥15 years was adjusted for gender and age in five-year bands. The 2010 census was used for age and gender adjustments.^29^ Analysis code is available at https://github.com/itidat/tropical-data-analysis-public. WASH variables were aggregated according to the WHO/UNICEF JMP definitions of improved water, sanitation and hygiene facilities. Because of the very small numbers of cases identified, no household-level disease association analyses were conducted.

## Results

### Population surveyed

In each EU, about 900 households (range: 898–1097) were included. In the nine EUs combined, 31,556 individuals aged ≥1 year were enumerated, of whom 27,962 were examined (Table 1).

**Table 1.** Number of people (≥1 years) enumerated, absent, refused and examined during trachoma surveys in Brazil, 2018–19.

Evaluation Unit	State	Enumerated	Absent at the time of visit	Long-term Absence*	Refused	Examined
Vale do Juruá	Acre	3579	213	151	17	3198
Sudoeste Amazonense	Amazonas	4334	309	242	28	3755
Norte de Roraima	Roraima	2932	192	125	31	2584
Nordeste Paraense	Pará	3958	237	179	37	3505
Leste Maranhense	Maranhão	3429	170	132	33	3094
Noroeste Cearense	Ceará	3089	146	124	40	2779
Sertão Pernambucano	Pernambuco	3191	306	10	67	2808
Sertão Alagoano	Alagoas	3827	277	164	113	3273
Vale São Franciscano da Bahia	Bahia	3217	115	112	24	2966

*Long-term absence is defined as those not likely to return to the house the evening immediately after the teams visit, for example those staying away for work, travel or hospitalisation

### Clinical signs

The number of children aged 1–9 years examined was 5,984 and 29 cases of TF (right and/or left eye) were found (Table 2).

**Table 2.** Prevalence of TF in children aged 1–9 years in selected evaluation units of Brazil, 2018–9.

Evaluation Unit	State	Number of 1–9-year-olds examined	Number of 1–9-year-olds with TF	Adjusted TF prevalence in 1–9-year-olds (%)*	95% Confidence Interval
Vale do Juruá	Acre	682	0	0.00	0.00–0.00
Sudoeste Amazonense	Amazonas	1045	8	0.67	0.13–1.47
Norte de Roraima	Roraima	591	5	0.62	0.10–1.35
Nordeste Paraense	Pará	764	9	0.96	0.29–1.88
Leste Maranhense	Maranhão	620	1	0.13	0.00–0.39
Noroeste Cearense	Ceará	554	3	0.58	0.00–1.50
Sertão Pernambucano	Pernambuco	466	2	0.20	0.00–0.51
Sertão Alagoano	Alagoas	648	0	0.00	0.00–0.00
Vale São Franciscano da Bahia	Bahia	614	1	0.08	0.00–0.24

*Adjusted to 2010 census data in one-year age bands.^29^

TF: trachomatous inflammation — follicular.

The prevalence of TF among children aged 1–9 years was low in all EUs surveyed. Prevalence ranged from 0.0% in Vale do Juruá to 1.0% in Nordeste Paraense (95%CI 0.3–1.9). All nine EUs had TF prevalence estimates well below the 5% threshold for elimination as a public health problem (Table 2).

The total number of people aged ≥15 years examined was 18,494, with 18 cases of TT found. Of these, 6 were known (meaning that the individuals reported having previously been offered management by a health worker) and 12 were unknown to the health system (Table 3).

**Table 3.** Prevalence of trachomatous trichiasis unknown to the health system in people aged ≥15 years in Brazil, 2018–9.

Evaluation Unit	State	Number of ≥15-year-olds examined	Number of TT cases unknown to the health system in ≥15-year-olds	Adjusted TT prevalence in ≥15-year-olds (%)*	95% Confidence interval
Vale do Juruá	Acre	2002	0	0.00	0.00–0.00
Sudoeste Amazonense	Amazonas	2226	0	0.00	0.00–0.00
Norte de Roraima	Roraima	1663	1	0.05	0.00–0.15
Nordeste Paraense	Pará	2312	0	0.00	0.00–0.00
Leste Maranhense	Maranhão	2035	0	0.00	0.00–0.00
Noroeste Cearense	Ceará	1971	7	0.22	0.06–0.44
Sertão Pernambucano	Pernambuco	2058	2	0.05	0.00–0.12
Sertão Alagoano	Alagoas	2230	0	0.00	0.00–0.00
Vale São Franciscano da Bahia	Bahia	1997	2	0.05	0.00–0.13

* Unknown to the health system (excluding cases for which management had previously been offered by a health worker); adjusted to 2010 census data for gender and age in five-year age bands.^29^

TT: trachomatous trichiasis.

**Figure 1.**
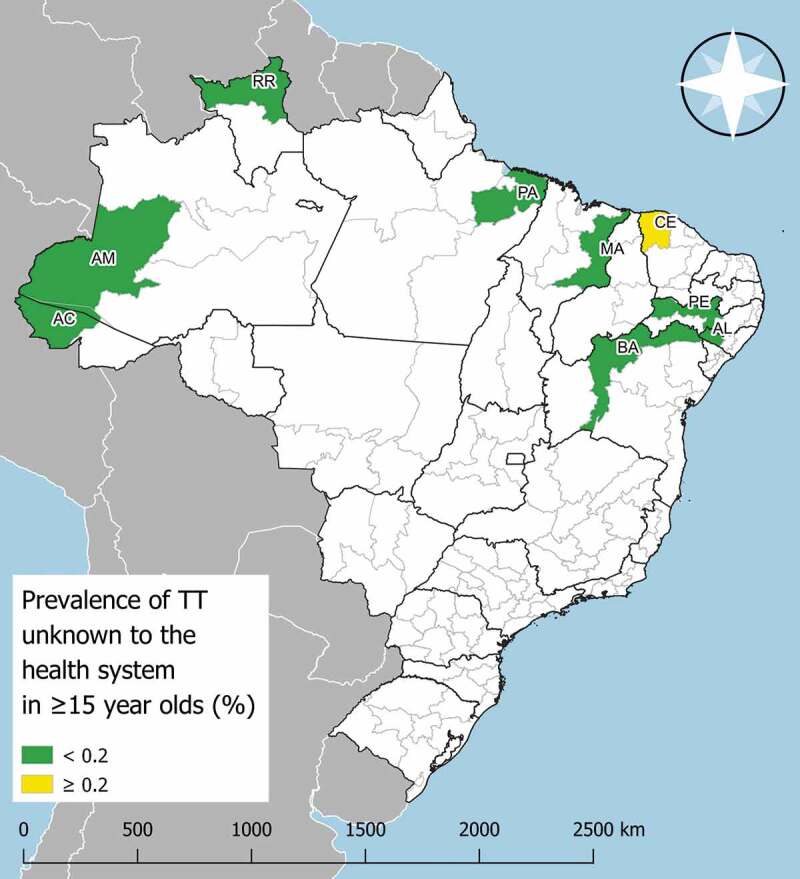
Trachomatous trichiasis (TT) unknown to the health system prevalence in ≥15-year-olds in evaluation units surveyed for trachoma in Brazil, 2018–9. The prevalence of trachomatous inflammation—follicular was <5% in all nine EUs. AC: Vale do Juruá; AM: Sudoeste Amazonense; RR: Norte de Roraima; PA: Nordeste Paraense; MA: Leste Maranhense; BA: Vale São Franciscano da Bahia; AL: Sertão Alagoano; PE: Sertão Pernambucano; CE: Noroeste Cearense. The boundaries and names shown and the designations used on this map do not imply the expression of any opinion whatsoever on the part of the World Health Organization or the Pan American Health Organization concerning the legal status of any country, territory, city or area or of its authorities, or concerning the delimitation of its frontiers or boundaries.

In eight of the nine EUs, the prevalence of TT unknown to the health system was below the elimination threshold of 0.2%.^15^ In the EU Noroeste Cearense, located in Ceara State, a prevalence of 0.22% was recorded (Figure 1).

### Household-level data

Data collected on household-level access to WASH, health care and education are presented by EU in Table 4. Although the proportions of households with piped water were very low, ranging from 1–23%, the proportions that had improved drinking water sources in the household were much higher, with values > 70% in three EUs. The percentages of households with improved water source less than a 30-minute journey from the house had a similar distribution (median: 66%, range: 43–86%). The proportion of households with ‘improved latrines’ according to WHO/UNICEF JMP criteria was variable between EUs: in three EUs, <40% of households had improved latrines, whereas in four other EUs, >80% of households had improved latrines.

**Table 4.** Indicators related to hygiene, sanitation conditions, health care and education.

Evaluation Unit	State	Number of households visited	% households with improved water source within 30-minute journey	% households with an improved latrine	% of households receiving monthly visits from community health workers	% of children from 5 to 9 years old who are in school
Vale do Juruá	Acre	901	71	36	63	99
Sudoeste Amazonense	Amazonas	903	76	36	77	98
Norte de Roraima	Roraima	898	66	86	36	95
Nordeste Paraense	Pará	898	63	62	61	100
Leste Maranhense	Maranhão	900	67	36	74	99
Noroeste Cearense	Ceará	900	86	92	64	99
Sertão Pernambucano	Pernambuco	1097	54	82	86	99
Sertão Alagoano	Alagoas	1087	43	77	65	98
Vale São Franciscano da Bahia	Bahia	900	47	88	66	99

Two-thirds of households surveyed received monthly visits from community health workers (median 65%, range: 36–86%). Only one EU had <60% of households visited monthly by community health workers (Norte de Roraima, 36%). There was a high school enrolment rate for children aged 5–9 years (median: 99%; range: 95–100%, Table 4).

## Discussion

The implementation of population-based prevalence survey protocols in Brazil, using internationally standardised and endorsed methodologies,^16,17^ allowed us to estimate as accurately as possible the trachoma prevalence in the at-risk non-indigenous Brazilian population. The findings of this study are highly encouraging in the context of Brazil’s fight to eliminate trachoma as a public health problem.

Although the selected survey areas did not cover the whole country, these nine EUs, formed within rural areas of high social and/or epidemiological risk, were chosen because they represented the non-indigenous locations of Brazil most likely to have trachoma. Given that the prevalence of TF was ≤1% in all nine EUs, we infer that the non-indigenous population of Brazil no longer has active trachoma at levels constituting a public health problem.

Among the possible explanations for the fact that trachoma appears to have disappeared in non-indigenous Brazil are the social and economic development of the country, with expansion of urban infrastructure and increased schooling of the young Brazilian population.^34^ Since the implementation of the National Program to Support the Collection of Rainwater and other Social Technologies (Cisterns Program) in 2003, focused on promoting access to water for human consumption, more than one million cisterns have been installed in semi-arid Brazil, with priority given to low-income rural families affected by drought or regular lack of water (http://mds.gov.br/assuntos/seguranca-alimentar/acesso-a-agua-1/programa-cisternas). According to data from the 2000 Demographic Census and the National Health Survey 2013, between these two time points, the percentage of households with piped water increased from 15.7 to 58.2% in the rural North, and from 16.5 to 55.8%, in the rural Northeast. The data presented here are consistent with the 2013 data: the proportion of households with an improved water source within 30 minutes of the house was 43–86% in the EUs surveyed. Regarding schooling, according to data from the National Household Sample Survey 2018, the schooling rate of 6–14-year-olds in 2018 was 99.3% for Brazil as a whole, 98.9% in the North and 99.2% in the Northeast (https://www.ibge.gov.br/estatisticas/sociais/trabalho/17270-pnad-continua.html?edicao=24772&t=resultados). In the surveys presented here, ≥95% of children aged 5–9 years reported regularly attending school.

In the late 1990’s, the Family Health Strategy was implemented as a national policy for primary care, giving priority to socially disadvantaged areas.^35^ Since its implementation, healthcare coverage has increased in all Brazilian regions and has been associated with a reduction in health problems treated under primary care. From 2007 to 2020, the proportion of people receiving regular visits from community health workers increased from 48.0 to 76.5% in Brazil, from 56.2 to 73.2%, in the North and from 75.0 to 86.5% in the Northeast (https://egestorab.saude.gov.br/). In our surveys, the poprotion of households reporting regular community health worker visits was >60% in 8/9 EUs; only in Norte de Roraima was a low percentage (36%) found. Strengthening of trachoma surveillance actions after the previous school-based surveys^27^ have led to the identification of endemic areas, improved access to diagnosis of trachoma, improved delivery of educational activities aimed at reducing the transmission of *C. trachomatis*,^36,37^ and improved access to antibiotics, including azithromycin, to treat trachoma and other conditions. All of these activities could have contributed to a substantial decline in trachoma prevalence in Brazil.

In the 2000s, government cash transfer programs, such as Bolsa Família, led to changes in income distribution and showed important impacts on health conditions,^38^ including a decrease in the incidence of some neglected tropical diseases.^39^ Thus, in addition to facilitating trachoma surveillance, public policies focused on decreasing social inequality may have contributed to reductions in trachoma in Brazil.

Only one EU (Noroeste Cearense) had a TT prevalence unknown to the health system ≥0.2% in ≥15-year-olds, and in that EU it was only 0.22% (95%CI 0.1–0.4%). One interpretation of this result would be that Noroeste Cearense still requires public health-level surgical service provision to achieve the TT elimination prevalence threshold, to be provided through collaboration between the health system of the state of Ceará, the Federal Ministry of Health, and relevant professional bodies. This could include measures to raise awareness among the general population about the availability of ophthalmic services, to identify cases and to ensure appropriate referral to certified surgeons, developing a collaborative plan to deal with the burden of trachomatous trichiasis in the adult population. In estimating the burden of a low prevalence condition, however, in which the confidence intervals of most estimates include zero, there is an inherent tendency for overestimation. The relative uncertainty of EU-level TT prevalence estimates is recognized,^40^ but is difficult to overcome using a frequentist statistical approach without the use of prohibitively large sample sizes. Further work, such as geospatial analysis of disease distribution should be undertaken on the data from these nine EUs before resources are deployed on a response in Noroeste Cearense that may or may not be required.

In addition to generating data on trachoma in each of the nine EUs, making it possible to assess an epidemiological situation of trachoma in Brazil, these surveys were valuable in requiring standardised training for trachoma diagnosis in accordance with WHO recommendations. Over 30 examiners from different states were trained and certified by Tropical Data.^30^ Capacity was also developed in Brazil for conducting population-based surveys using rigorous, internationally standardised approaches. Teams were able to complete surveys in EUs efficiently; on average, it took 15 days to survey an EU even in remote and hard-to-reach areas. This combined capacity can be deployed in the future should concerns about trachoma re-emerge, and could also be applied to surveys focussed on other health conditions.

Individuals enrolled in the survey benefitted from early detection of trachoma, making it possible to treat TF cases and family contacts at the time of the research, and referral of identified cases of TT to ophthalmic services. Moreover, the findings on access to WASH provide important supplementary information for public bodies looking to overcome social inequality.^41^

Indigenous communities were not included in this study. Future surveys are being planned in those populations, to help complete the mapping of trachoma prevalence in Brazil. In the meantime, Brazilian health authorities will work closely with WHO to ensure appropriate approaches are deployed to progress the agenda of elimination of trachoma as a public health problem.

## Conclusions

The prevalence of TF was well below the target for elimination as a public health problem in all non-indigenous surveyed EUs. Because these EUs were selected to represent the highest-risk non-indigenous areas of the country, TF prevalence is unlikely to be ≥5% in non-indigenous populations elsewhere. In one EU, the prevalence of TT was above the target threshold for elimination. Further investigation and possibly improvement in TT surgical provision are required in that EU.

## Supplementary Material

Supplemental Material
