# Supplementary material for: Population Prevalence of Trachoma in Nine Rural Non-Indigenous Evaluation Units of Brazil
Source: Ophthalmic Epidemiol. 2021 Oct 29;30(6):561–70. doi: 10.1080/09286586.2021.1941127 (PMC10581672; doi:10.1080/09286586.2021.1941127)
Supplement: Supplemental Material [file IOPE_A_1941127_SM3759.docx]

**Prevalência de tracoma em nove unidades de avaliação rurais na população não indígena do Brasil**

Célia Landmann Szwarcwald*, Maria de Fátima Costa Lopes, Paulo Roberto Borges de Souza Junior, Daniela Vaz Ferreira Gómez, Expedito José de Albuquerque Luna, Wanessa da Silva de Almeida, Giseli Nogueira Damacena, Joana da Felidade Ribeiro Favacho, Paulo Germano de Frias, Robert Butcher, Sarah Boyd, Ana Bakhtiari, Rebecca Willis, Cristina Jimenez, Emma Harding-Esch, Martha Idalí Saboyá-Díaz, Anthony W. Solomon.

*** Autor correspondente**

**Afiliações**

CLS, PRBSJ, WSA, GND: Instituto de Comunicação e Informação Científica e Tecnológica em Saúde, Fundação Oswaldo Cruz, Fundação Oswaldo Cruz, Rio de Janeiro, Brasil.

MFCL, DVFG: Coordenação de Vigilância de Zoonoses e Doenças Transmissíveis por Vetores, Departamento de Imunização e Doenças Transmissíveis, Secretaria de Vigilância em Saúde, Ministério da Saúde, Brasília, Brasil

EJAL: Departamento de Medicina Preventiva, Escola de Medicina, Universidade de São Paulo, São Paulo, Brasil

JFRF: Insituto Evandro Chagas, Secretaria de Vigilância em Saúde, Ministério da Saúde, Belém-Pará, Brasil

PGF: Instituto de Medicina Integral Professor Fernando Figueira (IMIP), Recife, Brasil

RB, EHE: London School of Hygiene & Tropical Medicine, London, UK

SB, AB, RW: International Trachoma Initiative, Task Force for Global Health, Atlanta, USA

CJ: Sightsavers International, Haywards Heath, UK

AWS: Department of Control of Neglected Tropical Diseases, World Health Organization, Geneva, Switzerland

MISD: Neglected, Tropical, and Vector-Borne Diseases Unit, Communicable Diseases and Environmental Determinants of Health Department, Pan American Health Organization (PAHO), Washington D.C., USA

**Título corrente**

Prevalência de tracoma no Brasil

**Contagem de palavras**

4.215

**Resumo**

**Propósito:** Para avaliar a prevalência atual do tracoma na população não indígena brasileira, foram realizados inquéritos em áreas supostamente de maior risco à doença.

**Métodos:** Considerando os municípios anteriormente endêmicos, foram selecionadas nove mesorregiões como Unidades de Avaliação (UA). Em cada UA, foram selecionados 30 setores rurais e 30 domicílios por setor. Os residentes dos domicílios foram examinados quanto à inflamação tracomatosa folicular (TF) e triquíase tracomatosa (TT). Foram coletados dados sobre acesso à água, saneamento, higiene e educação.

**Resultados:** Foram examinados 27.962 indivíduos nas 9 UA. A prevalência de TF ajustada por idade em crianças de 1 a 9 anos foi <5% em todas as UA. A prevalência de TT desconhecida para o sistema de saúde em pessoas de 15 anos ou mais foi <0,2% em oito UA, e 0,22% em apenas uma. A mediana da proporção de domicílios com acesso à fonte de água tratada foi 66%. O percentual de crianças de 5 a 9 anos que frequentavam escola foi maior que 99%.

**Conclusões:** A prevalência de TF por UA ficou bem abaixo da meta de eliminação do tracoma como problema de saúde pública em todas as UA, sendo improvável que a prevalência de TF seja ≥5% em outras áreas de população não indígena. Em apenas uma UA, a prevalência de TT ficou acima do limite crítico de eliminação. Investigações adicionais e, possivelmente, melhorias no acesso ao tratamento cirúrgico de TT são necessárias nessa UA.

**Palavras-chave**

Tracoma; triquíase; Brasil; prevalência; doenças tropicais negligenciadas; eliminação; WASH; Tropical Data.

**Introdução**

O tracoma é uma doença inflamatória crônica do olho causada pela bactéria intracelular *Chlamydia trachomatis*. O tracoma ativo (inflamatório) é caracterizado pela formação de folículos conjuntivais, que desaparecem espontaneamente quando a infecção é eliminada. Acredita-se que o *C. trachomatis* ocular seja transmitido principalmente de pessoa para pessoa pela transferência de secreções oculares e nasais durante o contato direto entre indivíduos, por meio de moscas que pousam nos olhos ou em fômites, como toalhas de rosto ou fronhas ^1–4^. Assim, o tracoma afeta desproporcionalmente comunidades pobres, onde a falta de recursos limita a higiene pessoal e o saneamento ^5–7^. Infecções repetidas levam a cicatrizes conjuntivais que podem, em casos graves, causar entrópio (pálpebra com a margem virada para dentro do olho) e triquíase (cílios em posição defeituosa tocando o globo ocular). Por sua vez, o atrito com o globo ocular ocasiona ulcerações na córnea, que reduzem a acuidade visual em diferentes níveis, podendo levar à cegueira ^8^. Acredita-se que muitas ocorrências de reinfecção sejam necessárias para o desenvolvimento de cicatrizes conjuntivais significativas ^9^, de modo que o tracoma representa uma ameaça à visão apenas quando a *C. trachomatis* ocular ocorre em crianças, sendo incomum a ocorrência de casos isolados ^10^.

O provável impacto do tracoma na saúde pública é avaliado, geralmente, utilizando-se o sistema simplificado de classificação da Organização Mundial da Saúde (OMS). Os cinco sinais do tracoma foram padronizados pela OMS, e abrangem os estágios inicial (inflamação tracomatosa - folicular [TF], inflamação tracomatosa - intensa [TI]) e tardio (cicatrização tracomatosa [TS], triquíase tracomatosa [TT], opacidade da córnea [CO]) de tracoma ^11,12^.

Em 2010, o tracoma foi considerado responsável por prejuízos visuais em 1,4 milhões de pessoas, das quais 450.000 apresentavam cegueira irreversível ^13^. Em março de 2019, estimou-se que 142 milhões de pessoas viviam em áreas endêmicas em todo o mundo ^14^. A OMS e seus parceiros têm como alvo a eliminação global do tracoma como um problema de saúde pública. Para declarar a eliminação, os países devem atender a três critérios: (1) prevalência de TF entre crianças de 1 a 9 anos <5% em áreas anteriormente endêmicas, (2) prevalência de TT desconhecida pelo sistema de saúde em pessoas com idade ≥ 15 anos <0,2% em áreas anteriormente endêmicas e (3) ter uma estratégia definida para identificar e gerenciar casos incidentes de TT ^15^. Nesse sentido, diretrizes de melhores práticas foram publicadas para apoiar os países a estimar a prevalência em áreas supostamente endêmicas ^16–19^.

O modelo de atenção para o controle do tracoma iniciou-se no Brasil a partir do ano de 1923 e se manteve até o ano de 1998, com a característica de ser centralizado, desenvolvido pelo Governo Federal, por meio de campanhas verticais. As atividades de vigilância e controle do tracoma no Brasil sofreram um decréscimo a partir da década de 1970, apesar de existirem estados com alta prevalência, em especial na região Norte, conforme inquérito de prevalência realizado no período de 1974 a 1976 ^20^. Entretanto, pelo fato de o tracoma ter sido considerado erradicado no estado de São Paulo na década de 1970, essa situação foi assumida como verdadeira para todo o país. A doença foi, então, negligenciada, perdendo a importância nas práticas de vigilância do Ministério da Saúde ^20^. No início dos anos 2000, para conhecer a situação do tracoma entre crianças, o Ministério da Saúde (MS) realizou uma pesquisa em uma amostra de escolas localizadas em 1.514 municípios com alta proporção de pessoas com precárias condições de vida. A pesquisa em escolares incluiu crianças indígenas que, reconhecidamente, são expostas ao maior risco ^21–23^. Todos os casos encontrados no inquérito foram tratados com antibiótico, com vistas ao controle da doença e à redução da transmissão de maneira pró-ativa ^24^. Em 7 dos 19 (37%) estados pesquisados, foram encontradas prevalências superiores a 5,0% ^25–27^.

Os resultados do inquérito deram visibilidade ao tracoma nos anos 2000, fortalecendo as atividades de vigilância e controle do tracoma no Brasil ^28^. Os casos encontrados nas atividades de vigilância e/ou campanhas, passaram a ser notificados no Sistema de Informações de Agravos de Notificação (SINAN) e monitorados continuamente. A atual estratégia de eliminação do tracoma no Brasil consiste na busca ativa de casos e rastreamento de contatos, cujo objetivo é reduzir a transmissão da infecção em áreas endêmicas por meio do tratamento com antibiótico de indivíduos com tracoma ativo e seus contatos ^24^.

Dados do SINAN de quase 1.000 municípios que realizaram atividades de vigilância de tracoma de 2008 a 2016 relatam uma queda acentuada na proporção de casos de tracoma encontrados. No entanto, como o tracoma afeta desproporcionalmente comunidades pobres, onde o acesso aos serviços públicos de saúde é precário, metodologias de amostragem que não são de base populacional, como amostragem de escolas ou sistemas de notificação do tracoma, correm o risco de apresentar viés. Diante dessa limitação, duas pesquisas domiciliares foram realizadas, posteriormente, nos estados de Pernambuco e Tocantins em 2014 e 2015 ^5^. No entanto, essas pesquisas não foram planejadas de acordo com os padrões internacionais atuais; em particular, os examinadores (classificadores) não foram padronizados como os de outros programas ^17^. Por esses motivos, e pelo fato de terem sido realizadas em apenas dois estados, considerou-se que seria necessário conduzir estudos com uma abrangência geográfica maior e que seguissem os padrões metodológicos dos protocolos da OMS ^16^ para apoiar uma futura declaração de eliminação do tracoma como um problema de saúde pública no Brasil.

Por ser o primeiro inquérito a ser realizado no Brasil seguindo o padrão metodológico da OMS, o objetivo deste estudo foi avaliar se os critérios para eliminação do tracoma como problema de saúde pública foram alcançados na população não indígena brasileira em nove áreas supostamente de risco ao tracoma. No presente artigo, descrevem-se a metodologia utilizada e os resultados obtidos na pesquisa.

**Materiais e métodos**

Desenho do estudo

Estudo observacional de corte transversal realizado em 2018-2019 para estimar a prevalência de TF em crianças de 1 a 9 anos de idade e a prevalência de TT na população de 15 anos ou mais de idade em áreas supostamente endêmicas. O inquérito seguiu as recomendações da OMS, com base no protocolo do GTMP ^16,17,19^, aperfeiçoado pelo Tropical Data ([www.tropicaldata.org](http://www.tropicaldata.org)).

O projeto foi desenvolvido pela Fundação Oswaldo Cruz e pela Secretaria de Vigilância em Saúde do Ministério da Saúde (SVS/MS) e revisto e implementado com o suporte do Tropical Data. O projeto foi aprovado pela Comissão de Ética em Pesquisa da Fundação Oswaldo Cruz (Parecer nº. 2.742.820) e da Organização Pan Americana da Saúde (Ref. No. 2018-06-0045). O apoio ao levantamento de dados foi aprovado pelo Comitê de Ética Observacional da London School of Hygiene & Tropical Medicine (16105).

Definição e seleção das unidades de avaliação

O Brasil é dividido territorialmente pelo Instituto Brasileiro de Geografia e Estatística (IBGE) em diferentes áreas geográficas: macrorregiões, mesorregiões e microrregiões (que não são unidades político-administrativas); e estados e municípios (que são unidades político-administrativas). À época dos censos demográficos, os municípios são subdivididos em setores censitários, que são classificados como rurais ou urbanos e indígenas ou não indígenas.

Os dados de inquéritos de prevalência e ações de busca ativa de casos realizadas em alguns Distritos Sanitários Especiais Indígenas apontam para níveis altos de prevalência de tracoma na população indígena, superiores aos da população não indígena ^27,28^. Contudo, as características demográficas da população indígena, o espalhamento geográfico das aldeias indígenas no território brasileiro, e as especificidades relacionadas à organização dos serviços de saúde e ao processo de aprovação da pesquisa, que necessita de aprovação pela Comissão Nacional de Ética em Pesquisa e autorização da Fundação Nacional do Índio para entrada em terras indígenas de todos os membros da equipe, nos levaram a dividir o projeto em dois, o primeiro na população não indígena e o segundo na população indígena, a ser desenvolvido no próximo ano.

Para a realização do inquérito na população não indígena brasileira, foram selecionadas nove mesorregiões homogêneas para compor as Unidades de Avaliação do tracoma no território nacional - áreas compostas de setores censitários rurais não indígenas, de risco ao tracoma, compondo regiões com população rural de 100 até 250 mil habitantes. Para tal, foram considerados os municípios anteriormente endêmicos de maior risco ao tracoma, definidos como os que apresentaram prevalências de TF superiores a 10% no inquérito realizado em escolares de 2002 a 2008 ^27^ ou grandes proporções de casos de TF encontrados nas ações de vigilância de 2008 a 2016. Para definir o risco social, foram considerados indicadores de pobreza e de saneamento: (1) valor do rendimento nominal médio mensal das pessoas de 10 anos ou mais de idade menor do que 25% do salário mínimo; (2) proporção de domicílios com abastecimento de água da rede geral menor do que 30%. Com base nestes critérios, foram escolhidas oito mesorregiões com alto risco ao tracoma nos seguintes estados: Acre, Amazonas, Roraima, Pará, Maranhão, Ceará, Alagoas e Pernambuco, contendo pelo menos um município anteriormente endêmico e o maior número de setores censitários não indígenas de risco social entre todas as mesorregiões do estado. Adicionalmente, para inclusão de uma Unidade de Avaliação silenciosa, selecionou-se uma mesorregião de muita pobreza no estado da Bahia, mas sem nenhum município com prevalência notificada de TF maior ou igual a 10%.

Em cada Unidade de Avaliação, o plano de amostragem foi em dois estágios. No primeiro estágio, foram selecionadas 30 localidades rurais não indígenas, sistematicamente, com probabilidade proporcional ao tamanho da população residente na localidade, de acordo com os dados do censo demográfico de 2010 ^29^. No segundo estágio do plano de amostragem, cada localidade foi mapeada e subdividida em segmentos de 30 domicílios vizinhos, aproximadamente, e, em cada localidade, foi selecionado um segmento por amostragem aleatória simples. A pesquisa foi realizada nos 30 domicíilios do segmento selecionado, totalizando 900 domicílios em cada UA.

Trabalho de campo

O trabalho de campo iniciou em setembro de 2018 e foi realizado em três UA até dezembro de 2018. Recomeçou em agosto de 2019 e finalizou em outubro, sendo realizado nas demais 6 UA. Cada equipe de campo era composta por um registrador, um examinador de tracoma e um agente de saúde local, que acompanhava a equipe para facilitar o trabalho de campo nos locais selecionados. Todos os membros da equipe foram padronizados nos procedimentos do protocolo. Os examinadores e registradores de tracoma foram padronizados de acordo com as recomendações da OMS e dos manuais do Tropical Data ^30^.

Adicionalmente, as equipes de campo foram supervisionadas (dois supervisores) e dois oftalmologistas apoiaram a avaliação e encaminhamento de casos de TT aos serviços de saúde.

Para a abordagem nos domicílios, foi feito contato com a pessoa responsável e com os moradores para explicar o estudo, objetivos, procedimentos, e a importância de participação na pesquisa. Todos os moradores de um ano ou mais de idade foram convidados a participar da pesquisa. Após esta etapa, cada morador adulto (18 anos ou mais) dava o seu consentimento em participar da pesquisa após a leitura do Termo de Consentimento Livre e Esclarecido, bem como consentia na participação dos menores de 18 anos sob a sua responsabilidade. Para os moradores de 7 a 17 anos de idade, foi apresentado o Termo de Assentimento Livre e Esclarecido.

Após o consentimento em participar da pesquisa, detectavam-se as coordenadas GPS do domicílio, e uma entrevista era realizada com um informante-chave do domicílio, sobre a situação de saneamento ambiental e informações referentes à fonte da água usada pelos residentes no domicílio para beber e se lavar (variáveis WASH). Esse instrumento foi elaborado a partir da tradução do questionário domiciliar utilizado no Tropical Data, adaptado do questionário do Programa de Monitoramento Conjunto da OMS / UNICEF (JMP), e modificado (com mínimas alterações) para se adequar à realidade brasileira sob aprovação de especialistas do Tropical Data. Durante a entrevista com o informante-chave do domicílio, perguntava-se, adicionalmente, se o domicílio recebia visitas mensais de agentes comunitários de saúde.

Na segunda parte da entrevista, foram coletadas informações sociodemográficas de todos os moradores examinados com um ano ou mais de idade. Às crianças de 5 a 9 anos de idade, foi perguntado se elas frequentavam regularmente a escola. No caso de moradores ausentes no domicílio, foi realizada uma visita de retorno no final do dia.

As entrevistas foram feitas pelo registrador com a utilização do aplicativo de *smartphone* Tropical Data. O registrador era responsável pelo armazenamento dos dados no *smartphone*, bem como pela transmissão das informações via internet para a plataforma Tropical Data. Os dados foram criptografados e transmitidos para servidores seguros para limpeza, armazenamento e análise pela equipe de dados da Tropical Data, em colaboração com a Fiocruz e o Ministério da Saúde. Esse sistema permitiu o monitoramento do trabalho de campo em tempo real e da aderência aos protocolos acordados previamente.

Exame clínico

A classificação clínica foi conduzida por examinadores que completaram com sucesso o programa de treinamento dos Tropical Data e alcançaram uma pontuação kappa ≥0,7 em comparação com o treinador certificado de graduação do Tropical Data na classificação de 50 crianças em idade escolar, das quais pelo menos 10% tinham TF ^30^. As crianças das escolas foram pré-selecionadas na semana anterior à alcançaram uma pontuação para garantir que houvesse casos suficientes de TF, de acordo com as diretrizes da Tropical Data. O exame externo de ambos os olhos de todos os residentes com idade ≥ 1 ano que consentiram em participar da pesquisa foi realizado, usando lupas de ampliação de 2,5 ×. A presença ou ausência de TT, TF e TI, definida de acordo com o sistema simplificado de classificação da OMS ^11^, foi registrada para cada olho examinado.

Na detecção de algum caso de TF ou TI, o examinador dava ciência ao indivíduo ou ao responsável pelo menor infectado e oferecia tratamento medicamentoso gratuito com azitromicina. Crianças com TF ou TI e seus familiares receberam uma dose do antibiótico recomendado pela OMS de acordo com a idade e o peso ^31^, conforme regulamentado pela Secretaria de Vigilância em Saúde, Ministério da Saúde ^32^.

No caso de detecção de TT, a conjuntiva tarsal superior era examinada quanto a sinais de TS e foram feitas questões adicionais para identificar se o indivíduo havia ou não recebido tratamento prévio (ou seja, se ele era "desconhecido do sistema de saúde") ^33^.

As informações de contato dos casos de TT foram registradas e enviadas às autoridades de saúde locais - às Secretarias Municipais de Saúde (SMS) e às Secretarias Estaduais de Saúde (SES) - responsáveis por encaminhá-las à avaliação oftalmológica e, eventualmente, à cirurgia, se necessário.

As equipes foram capacitadas, igualmente, para o desenvolvimento de ações de educação em saúde específicas para o controle e prevenção do tracoma. Essas ações educativas têm como foco a melhoria da higiene pessoal, com ênfase no estímulo à lavagem facial e outras formas de prevenção da doença.

Análise de dados

O plano complexo de amostragem foi considerado na análise dos dados e na estimação dos indicadores de eliminação do tracoma. As estimativas ajustadas de prevalência foram calculadas no software estatístico R usando os algoritmos do Tropical Data, que são baseados nos do GTMP ^17^ e produzem, no nível de UA, proporções ajustadas pela amostragem em conglomerados. A prevalência de TF em crianças de 1 a 9 anos de idade foi ajustada por idade. A prevalência de TT na população de 15 anos ou mais foi ajustada por sexo e faixas quinquenais de idade. Os ajustes por idade e sexo foram realizados utilizando-se os dados do Censo Demográfico de 2010 ^29^. O código da programação de análise está disponível em https://github.com/itidat/tropical-data-analysis-public. As variáveis de acesso à água, saneamento e higiene (WASH) foram agregadas de acordo com as categorias do JMP da OMS / UNICEF. Devido ao número muito pequeno de casos identificados, nenhuma análise de associação com as variáveis WASH no nível de domicílio foi realizada.

**Resultados**

Em cada Unidade de Avaliação, foram pesquisados cerca de 900 (898-1097) domicílios. No total das nove UA, foram enumerados 31.556 indivíduos um ano ou mais de idade, dos quais 27.962 foram examinados (Tabela 1).

Sinais clínicos

O número total de crianças de 1 a 9 anos de idade examinadas foi de 5.984 e 29 casos de TF foram encontrados (Tabela 2).

A prevalência de TF entre crianças de 1 a 9 anos de idade foi baixa em todas as UA pesquisadas. Os valores variaram de 0,0% no Vale do Juruá, no Acre, a 1,0%, no Nordeste Paraense, no Pará (IC95% 0,3-1,9). Ou seja, todas as nove UA mostraram prevalências significativamente inferiores ao limite crítico de 5% para eliminação como um problema de saúde pública (Tabela 2).

O número total de pessoas de 15 anos ou mais de idade examinadas foi de 18.494 e foram encontrados 18 casos de triquíase tracomatosa. Desses, 6 eram conhecidos e 12 eram desconhecidos pelo sistema de saúde (Tabela 3).

Em oito das nove UA, o indicador de prevalência de TT desconhecida pelo sistema de saúde foi inferior ao limite crítico de 0,2% ^15^. Apenas no Noroeste Cearense, região localizada no Ceará, foi encontrada uma prevalência de 0,22% (Figura 1).

Dados no nível domiciliar

Os indicadores relacionados às condições de higiene e saneamento, à assistência de saúde e educação estão apresentados na Tabela 4. Embora as proporções de domicílios com água canalizada tenham sido muito baixas, variando de 1% a 23%, os percentuais de domicílios com fonte de água potável no domicílio foram bem maiores, com valores superiores a 70% em três UA. Da mesma forma, os percentuais de domicílios com fonte de água adequada a menos de uma distância percorrida em 30 minutos apresentaram variação semelhante, com valores pouco menores (mediana: 66%, amplitude: 43–86%). A proporção de domicílios com “banheiro adequado” de acordo com os critérios do JMP da OMS / UNICEF apresentou variações importantes, com percentuais inferiores a 40% em três das UA e superiores a 80% em quatro UA.

Em relação à assistência de saúde, o percentual de domicílios que recebem visita mensal de agentes comunitários de saúde ficou, em geral, entre 60 e 70% (mediana de 65% e amplitude de variação 36-86%). Apenas a UA Norte de Roraima apresentou um valor bem abaixo da média (36%). Chama a atenção o grande percentual de crianças de 5 a 9 anos que estão na escola, atingindo quase 100% em todas as nove UA, com mediana de 99% e amplitude de variação 97-100% (Tabela 4).

**Discussão**

A realização do inquérito de prevalência de base domiciliar no Brasil, seguindo, pela primeira vez no Brasil, todas as recomendações do Projeto Global de Eliminação do Tracoma, utilizando metodologias padronizadas e endossadas internacionalmente ^16,17^, permitiu estimar com a maior precisão possível a prevalência do tracoma na população brasileira não indígena. Os resultados deste estudo são altamente encorajadores no contexto de eliminação do tracoma como problema de saúde pública no Brasil.

Embora as áreas selecionadas para o estudo não cubram a totalidade do país, as nove unidades de avaliação, formadas por áreas rurais de alto risco ao tracoma e de grande vulnerabilidade social, foram escolhidas para a realização do inquérito sob a suposição que essas áreas representam a situação do tracoma nas populações não indígenas expostas ao maior risco à doença no Brasil. Tendo em vista que as prevalências de TF se mostraram inferiores a 1% nas nove Unidades de Avaliação, inferimos que a população não indígena do Brasil não possui mais tracoma ativo em níveis que constituam um problema de saúde pública.

Entre as possíveis explicações para a nítida melhora na situação do tracoma no Brasil não indígena estão: o desenvolvimento social e econômico do País, com a ampliação do acesso à infraestrutura urbana e aumento da escolaridade da população jovem brasileira ^34^. Desde a implementação do Programa Nacional de Apoio à Coleta de Água da Chuva e outras Tecnologias Sociais (Programa de Cisternas), em 2003, dirigido à promoção de acesso à água para consumo humano, mais de um milhão de cisternas foram instaladas no Brasil semiárido, priorizando famílias rurais de baixa renda afetadas pela seca ou falta regular de água (http://mds.gov.br/assuntos/seguranca-alimentar/acesso-a-agua-1/programa-cisternas). Segundo dados do Censo Demográfico de 2000 e da Pesquisa Nacional de Saúde 2013, de 2000 a 2013, o percentual de domicílios com água encanada aumentou de 15,7 para 58,2% no Norte rural e de 16,5 para 55,8% no Nordeste rural. Os dados do presente estudo são consistentes com os dados da Pesquisa Nacional de Saúde 2013, sugerindo que o percentual de domicílios com acesso a uma fonte de água adequada variou entre 43-86% na UA pesquisada. Em relação à escolarização, segundo dados da Pesquisa Nacional por Amostra de Domicílios 2018, a taxa de escolarização de crianças de 6 a 14 anos em 2018 era de 99,3% para o Brasil, 98,9% no Norte e 99,2% no Nordeste ([https://www.ibge.gov.br/estatisticas/sociais/trabalho/17270-pnad-continua.html?edicao=24772&t=resultados](https://eur05.safelinks.protection.outlook.com/?url=https%3A%2F%2Fwww.ibge.gov.br%2Festatisticas%2Fsociais%2Ftrabalho%2F17270-pnad-continua.html%3Fedicao%3D24772%26t%3Dresultados&data=02%7C01%7C%7Cc47f3245b77d477c336908d812fb087d%7C84df9e7fe9f640afb435aaaaaaaaaaaa%7C1%7C0%7C637280217930285914&sdata=ArplpSBdFQAbYCLlfe8MhyZBGEsWeQYMyYiMIu43KnM%3D&reserved=0)). Nos resultados aqui apresentados, percentual maior ou igual a 95% das crianças de 5 a 9 anos de idade relataram frequentar regularmente a escola.

No final dos anos 90, a Estratégia de Saúde da Família foi implementada como uma política nacional de atenção primária, priorizando áreas socialmente desfavorecidas ^35^. Desde sua implementação, a cobertura aumentou em todas as regiões brasileiras e tem sido associada à redução diferencial de problemas de saúde passíveis de resolução com cuidados de atenção primária em saúde. De 2007 a 2020, a proporção de pessoas que recebem visitas regulares de agentes comunitários de saúde aumentou de 48,0% para 76,5%, no Brasil, de 56,2 para 73,2, no Norte, e de 75,0 para 86,5 no Nordeste (https://egestorab.saude.gov.br /). Os resultados deste estudo revelaram que o percentual de domicílios que tiveram visitas regulares de agentes comunitários de saúde foi superior a 60% em oito das nove UA pesquisadas. Apenas no Norte de Roraima, um percentual pequeno (36%) foi encontrado. O fortalecimento das ações de vigilância do tracoma após a pesquisa anterior nas escolas ^27^ permitindo identificar áreas endêmicas, junto à ampliação do acesso ao diagnóstico e ao tratamento pelo Programa de Saúde da Família, a expansão das ações educativas para a prevenção da transmissão de *C. trachomatis* ^36,37^, e o uso de antibióticos, incluindo a azitromicina, para tratar outros problemas de saúde contribuíram substancialmente para a redução do tracoma no Brasil.

Nos anos 2000, os programas governamentais de transferência de renda, como o bolsa-família, levaram a mudanças na distribuição de renda e mostraram impactos importantes nas condições de saúde ^38^, incluindo a diminuição da incidência de algumas doenças tropicais negligenciadas ^39^. Assim, além do fortalecimento da vigilância do tracoma, as políticas públicas com foco na diminuição das desigualdades sociais contribuíram, possivelmente, para a redução da endemicidade do tracoma na população brasileira.

Quanto às prevalências de TT, apenas uma das UA (Noroeste Cearense) apresentou prevalência de TT desconhecida para o sistema de saúde ≥0,2% em pessoas com mais de 15 anos de idade. Nesta UA, a prevalência foi de apenas 0,22% (IC95% 0,1-0,4%). Uma interpretação deste resultado é que o Noroeste Cearense ainda requer ampliar a detecção dos casos de TT e o acesso aos serviços cirúrgicos para atingir a meta de eliminação de triquíase não gerenciada pelo sistema de saúde, por meio da colaboração entre o sistema de saúde do estado do Ceará, o Ministério da Saúde e os órgãos competentes, além de profissionais de saúde. Isso pode incluir medidas para aumentar a conscientização da população em geral sobre a disponibilidade de serviços oftalmológicos, identificar casos e garantir encaminhamento adequado a cirurgiões certificados, desenvolvendo um plano colaborativo para lidar com a carga de triquíase tracomatosa na população adulta. Contudo, ao estimar a carga de uma condição de baixa prevalência, em que os intervalos de confiança da maioria das estimativas incluem zero, existe uma tendência inerente à superestimação. A incerteza relativa das estimativas de prevalência de TT no nível da UA é reconhecida ^40^, mas é uma questão difícil de ser solucionada utilizando uma abordagem estatística frequentista sem o uso de amostras proibitivamente grandes. Trabalhos adicionais, incluindo análise geoestatística, devem ser realizados sobre os dados dessas nove UA antes que os recursos sejam utilizados para uma resposta no Noroeste Cearense, que pode ou não ser necessária.

Além da obtenção de dados fidedignos sobre o tracoma em cada uma das nove Unidades de Avaliação, possibilitando avaliar a situação epidemiológica do tracoma no Brasil, a pesquisa foi exitosa em ampliar a capacitação padronizada para a detecção do tracoma de acordo com as recomendações da OMS. Mais de 30 examinadores de diferentes estados foram capacitados e certificados pelo Tropical Data ^30^. Uma contribuição adicional da pesquisa foi a capacitação de vários pesquisadores para a realização de inquéritos populacionais usando abordagens rigorosas e padronizadas internacionalmente. As equipes conseguiram concluir o trabalho de campo nas unidades de avaliação com eficiência, levando, em média, 15 dias para pesquisar uma UA, mesmo em áreas remotas e de difícil acesso. Essa capacidade combinada poderá ser empregada no futuro, caso preocupações sobre o tracoma reemerjam, ou poderá ser aplicada a pesquisas focadas em outros problemas de saúde.

Em termos de benefícios da pesquisa para a população, citam-se a detecção precoce do tracoma, possibilitando dar tratamento aos casos de TF e aos contatos familiares no momento da pesquisa, e o encaminhamento de todos os casos identificados de triquíase tracomatosa (TT) em adultos às unidades de referência oftalmológica. E ainda, os achados acerca da situação de higiene e saneamento fornecem subsídios importantes para as políticas públicas de superação das desigualdades sociais ^41^.

As comunidades indígenas não foram incluídas neste estudo, mas pesquisas estão sendo planejadas em distritos sanitários indígenas, para concluir o mapeamento da prevalência de tracoma no Brasil. Nesse interim, os pesquisadores brasileiros trabalharão em estreita colaboração com a OMS para garantir que abordagens apropriadas sejam usadas para avançar na agenda de eliminação do tracoma como um problema de saúde pública no país.

**Conclusões**

A prevalência de TF foi bem abaixo da meta de eliminação do tracoma como problema de saúde pública em todas as UA pesquisadas. Tendo em vista que as unidades de avaliação foram selecionadas para representar as áreas não indígenas de maior risco no país, é improvável que a prevalência seja superior a 5% em outras áreas não indígenas. Em apenas uma UA, a prevalência de TT foi superior ao limite da meta de eliminação. Nessa UA, maiores investigações são necessárias e, possivelmente, a expansão do acesso a cirurgias de TT.

**Financiamento**

Este projeto de pesquisa foi financiado pelo Ministério da Saúde do Brasil.

O financiamento de Tropical Data fundamentais foi fornecido pela International Trachoma Initiative; Sightsavers; e RTI International através do United States Agency for International Development (USAID) Act to End NTDs | East program. O conteúdo não reflecte necessariamente a opinião da USAID ou do Governo dos Estados Unidos.

Os autores são os únicos responsáveis pelas opiniões expressas neste artigo, que não representam necessariamente as opiniões, decisões ou políticas das instituições às quais estão afiliados. Além da contribuição dos autores, nenhum dos financiadores teve algum papel na concepção do projeto, na implementação ou análise ou interpretação dos dados, nas decisões sobre onde, como ou quando publicar em revistas científicas com revisão por pares ou na preparação do manuscrito.

**Declaração de interesse**

SB, AB e RW são funcionários da International Trachoma Initiative-The Task Force for Global Health, que recebe um orçamento operacional e fundos de pesquisa da Pfizer Inc., fabricantes de Zithromax® (azitromicina). A Pfizer doa Zithromax por meio da International Trachoma Initiative para fins de eliminação do tracoma, quando necessário. EHE recebe apoio salarial da International Trachoma Initiative. Os outros autores não relatam conflitos de interesse. MISD é funcionária da Organização Pan-Americana da Saúde (OPAS). AWS é membro da equipe da Organização Mundial da Saúde. Os autores são os únicos responsáveis pelas opiniões expressas neste artigo e não representam necessariamente as opiniões, decisões ou políticas das instituições às quais estão afiliados. Em qualquer reprodução deste artigo, não deve haver sugestão de que a OPAS, a OMS ou este artigo endossem qualquer organização, produto ou serviço específico. O uso de logotipos da OPAS ou OMS não é permitido. Este aviso deve ser preservado junto com o URL original do artigo.

**Referências**

1. Reilly LA, Favacho J, Garcez LM, Courtenay O. Preliminary evidence that synanthropic flies contribute to the transmission of trachoma-causing Chlamydia trachomatis in Latin America. *Cad Saude Publica*. 2007;23(7):1682-1688. doi:10.1590/S0102-311X2007000700020

2. Last A, Versteeg B, Abdurahman OS, et al. Detecting extra-ocular chlamydia trachomatis in a trachoma-endemic community in ethiopia: Identifying potential routes of transmission. *PLoS Negl Trop Dis*. 2020;14(3):18-32. doi:10.1371/journal.pntd.0008120

3. Robinson A, Bristow J, Holl M V., et al. Responses of the putative trachoma vector, musca sorbens, to volatile semiochemicals from human faeces. *PLoS Negl Trop Dis*. 2020;14(3):1-15. doi:10.1371/journal.pntd.0007719

4. Miller K, Pakpour N, Yi E, et al. Pesky trachoma suspect finally caught. *Br J Ophthalmol*. 2004;88(6):750-751. doi:10.1136/bjo.2003.038661

5. Gondim de Brito CM, Cavalcanti Barbosa C, Coelho de Andrade SM, et al. Household Survey of Trachoma among Children living in Pernambuco, Brazil. *Pathogens*. 2019;8(4):1-13.

6. Cruz AA V, Medina NH, Ibrahim MM, Souza RM, Gomes UA, Goncalves GFOR. Prevalence of trachoma in a population of the upper Rio Negro basin and risk factors for active disease. *Ophthalmic Epidemiol*. 2008;15(4):272-278. doi:10.1080/09286580802080090

7. Habtamu E, Wondie T, Aweke S, et al. Trachoma and Relative Poverty: A Case-Control Study. *PLoS Negl Trop Dis*. 2015;9(11):e0004228. doi:10.1371/journal.pntd.0004228

8. Taylor HR. *Trachoma: A Blinding Scourge from the Bronze Age to the Twenty-First Century*. East Melbourne: Centre for Eye Research, Australia; 2008.

9. Gambhir M, Basáñez M-G, Burton MJ, et al. The development of an age-structured model for trachoma transmission dynamics, pathogenesis and control. *PLoS Negl Trop Dis*. 2009;3(6):e462. doi:10.1371/journal.pntd.0000462

10. Lansingh VC. Trachoma. *BMJ Clin Evid*. 2016;2016.

11. Thylefors B, Dawson CR, Jones BR, West SK, Taylor HR. A simple system for the assessment of trachoma and its complications. *Bull World Health Organ*. 1987;65(4):477-483.

12. Solomon AW, Kello AB, Bangert M, et al. The simplified trachoma grading system, amended. *Bull World Health Organ*. 2020;98(10):645-724.

13. Bourne RA, Stevens GA, White RA, et al. Causes of vision loss worldwide, 1990-2010: a systematic analysis. *Lancet Glob Health*. 2013;1(6):e339-49.

14. World Health Organization. WHO Alliance for the Global Elimination of Trachoma by 2020: progress report on elimination of trachoma, 2018. *Wkly Epidemiol Rec*. 2019;(29):317-328.

15. World Health Organization. *Validation of Elimination of Trachoma as a Public Health Problem (WHO/HTM/NTD/2016.8).* Geneva, Switzerland; 2016.

16. World Health Organization. *Design Parameters for Population-Based Trachoma Prevalence Survey (WHO/HTM/NTD/PCT/2018.07).* Geneva, Switzerland; 2018.

17. Solomon AW, Pavluck AL, Courtright P, et al. The Global Trachoma Mapping Project: Methodology of a 34-Country Population-Based Study. *Ophthalmic Epidemiol*. 2015;22(3):214-225.

18. World Health Organization. Tropical Data: a WHO-led initiative to help national programmes collect and do more with their data. Web Release. https://www.who.int/trachoma/news/News_Trachoma_Tropical_Data_launch/en/. Published 2016. Accessed June 20, 2007.

19. Solomon AW, Willis R, Pavluck AL, et al. Quality Assurance and Quality Control in the Global Trachoma Mapping Project. *Am J Trop Med Hyg*. 2018;99(4):858-863. doi:10.4269/ajtmh.18-0082

20. Schellini A, Sousa R. Trachoma: still being an important blinding disease. *Rev Bras Oftalmol*. 2012;71(3):199-204.

21. Paula JS, Medina NH, Cruz AA V. Trachoma among the Yanomami Indians. *Brazilian J Med Biol Res*. 2002;35(10):1153-1157. doi:10.1590/s0100-879x2002001000007

22. Freitas HS de A, Medina NH, Lopes M de FC, et al. Trachoma in Indigenous Settlements in Brazil, 2000–2008. *Ophthalmic Epidemiol*. 2016;23(6):354-359. doi:10.3109/09286586.2015.1131305

23. Cruz AAV. Blinding trachoma among Maku Indians of the upper Rio Negro: A neglected public health problem. *Arq Bras Oftalmol*. 2017;80(3):v-vi. doi:10.5935/0004-2749.20170035

24. Brazil Ministry of Health., Health Surveillance Secretariat. *Manual de Vigilância Do Tracoma e Sua Eliminação Como Causa de Cegueira.* 2nd ed. Brasilia, Brazil; 2014.

25. Medina NH, Lopes M de F, Durkin SR, et al. Survey of trachoma within school students in the state of Roraima, Brazil. *Ophthalmology*. 2011;118(10):1938-1943. doi:10.1016/j.ophtha.2011.02.047

26. Barbarini Ferraz LC, Schellini SA, Padovani CR, et al. Prevalence of trachoma among school children in Bauru - Sao Paulo State, Brazil. *Arq Bras Oftalmol*. 2010;73(5):433-437. doi:10.1590/s0004-27492010000500009

27. Lopes MDFC, Luna EJDA, Medina NH, et al. Prevalence of trachoma in Brazilian schoolchildren. *Rev Saude Publica*. 2013;47(3):451-459. doi:10.1590/S0034-8910.2013047003428

28. Luna EJ de A, Lopes M de FC, Medina NH, Favacho J, Cardoso MRA. Prevalence of Trachoma in Schoolchildren in Brazil. *Ophthalmic Epidemiol*. 2016;23(6):360-365. doi:10.1080/09286586.2016.1244274

29. Brazilian Institute of Geography and Statistics (IBGE). *Brazil Demographic Census 2010.* Rio de Janeiro, Brazil: Brazilian Institute of Geography and Statistics (IBGE); 2012.

30. Courtright P, MacArthur C, Macleod C, et al. *Tropical Data: Training System for Trachoma Prevalence Surveys (Version 2).* London, UK; 2017.

| **Unidade de Avaliação** | **Estados** | **Enumerados** | **Ausentes na hora da visita** | **Ausência prolongada** | **Recusas** | **Examinados** |
| --- | --- | --- | --- | --- | --- | --- |
| Vale do Juruá | Acre | 3579 | 213 | 151 | 17 | 3198 |
| Sudoeste Amazonense | Amazonas | 4334 | 309 | 242 | 28 | 3755 |
| Norte de Roraima | Roraima | 2932 | 192 | 125 | 31 | 2584 |
| Nordeste Paraense | Pará | 3958 | 237 | 179 | 37 | 3505 |
| Leste Maranhense | Maranhão | 3429 | 170 | 132 | 33 | 3094 |
| Noroeste Cearense | Ceará | 3089 | 146 | 124 | 40 | 2779 |
| Sertão Pernambucano | Pernambuco | 3191 | 306 | 10 | 67 | 2808 |
| Sertão Alagoano | Alagoas | 3827 | 277 | 164 | 113 | 3273 |
| Vale São Franciscano da Bahia | Bahia | 3217 | 115 | 112 | 24 | 2966 |

**Tabela 1.** Número de pessoas pesquisadas, ausentes, recusas e examinadas em unidades de avaliação selecionadas do Brasil, 2018-19

| **Unidade de Avaliação** | **Estado** | **Número de crianças de 1-9 anos examinadas** | **Número de casos de TF em crianças de 1-9 anos** | **Prevalência ajustada de TF entre crianças de 1-9 anos (%)*** | **Intervalo de 95% de Confiança** |
| --- | --- | --- | --- | --- | --- |
| Vale do Juruá | Acre | 682 | 0 | 0.00 | 0.00 – 0.00 |
| Sudoeste Amazonense | Amazonas | 1045 | 8 | 0.67 | 0.13 – 1.47 |
| Norte de Roraima | Roraima | 591 | 5 | 0.62 | 0.10 – 1.35 |
| Nordeste Paraense | Pará | 764 | 9 | 0.96 | 0.29 – 1.88 |
| Leste Maranhense | Maranhão | 620 | 1 | 0.13 | 0.00 – 0.39 |
| Noroeste Cearense | Ceará | 554 | 3 | 0.58 | 0.00 – 1.50 |
| Sertão Pernambucano | Pernambuco | 466 | 2 | 0.20 | 0.00 – 0.51 |
| Sertão Alagoano | Alagoas | 648 | 0 | 0.00 | 0.00 – 0.00 |
| Vale São Franciscano da Bahia | Bahia | 614 | 1 | 0.08 | 0.00 – 0.24 |

* Ajustada pela população de 1 a 9 anos do Censo Demográfico, 2010 em grupos de idade de 1 ano ^29^.

TF: inflamação tracomatosa - folicular

**Tabela 2.** Prevalência de TF em crianças de 1–9 anos de idade em unidades de avaliação selecionadas do Brasil, 2018–9.

| **Unidade de Avaliação** | **Estado** | **Número de pessoas examinadas de 15 anos ou mais** | **Número de** **casos de TT de 15 anos ou mais desconhecidos pelo sistema de saúde** | **Prevalência ajustada de TT* entre as pessoas de 15 anos ou mais(%)*** | **Intervalo de 95%de Confiança** |
| --- | --- | --- | --- | --- | --- |
| Vale do Juruá | Acre | 2002 | 0 | 0.00 | 0.00 – 0.00 |
| Sudoeste Amazonense | Amazonas | 2226 | 0 | 0.00 | 0.00 – 0.00 |
| Norte de Roraima | Roraima | 1663 | 1 | 0.05 | 0.00 – 0.15 |
| Nordeste Paraense | Pará | 2312 | 0 | 0.00 | 0.00 – 0.00 |
| Leste Maranhense | Maranhão | 2035 | 0 | 0.00 | 0.00 – 0.00 |
| Noroeste Cearense | Ceará | 1971 | 7 | 0.22 | 0.06 – 0.44 |
| Sertão Pernambucano | Pernambuco | 2058 | 2 | 0.05 | 0.00 – 0.12 |
| Sertão Alagoano | Alagoas | 2230 | 0 | 0.00 | 0.00 – 0.00 |
| Vale São Franciscano da Bahia | Bahia | 1997 | 2 | 0.05 | 0.00 – 0.13 |

* Desconhecidos pelo sistema de saúde (excluindo casos para os quais o manejo foi oferecido por profissional de saúde); ajustada pela população do Censo Demográfico de 2010 por sexo e faixa quinquenal de idade ^29^.

TT: triquíase tracomatosa

**Tabela 3.** Prevalência de triquíase tracomatosa desconhecida pelo sistema de saúde em pessoas de 15 anos ou mais de idade em unidades de avaliação selecionadas do Brasil, 2018–9.

| **Unidade de Avaliação** | **Estado** | **Número de residências visitadas** | **% domicílios com fonte de água adequada para beber a 30 minutos ou menos** | **% de domicílios com latrinas consideradas adequadas^1^** | **% de domicílios que recebem visitas mensais de agentes de saúde** | **% de crianças de 5 a 9 anos que frequentam escola** |
| --- | --- | --- | --- | --- | --- | --- |
| Vale do Juruá | Acre | 901 | 71 | 36 | 63 | 99 |
| Sudoeste Amazonense | Amazonas | 903 | 76 | 36 | 77 | 98 |
| Norte de Roraima | Roraima | 898 | 66 | 86 | 36 | 95 |
| Nordeste Paraense | Pará | 898 | 63 | 62 | 61 | 100 |
| Leste Maranhense | Maranhão | 900 | 67 | 36 | 74 | 99 |
| Noroeste Cearense | Ceará | 900 | 86 | 92 | 64 | 99 |
| Sertão Pernambucano | Pernambuco | 1097 | 54 | 82 | 86 | 99 |
| Sertão Alagoano | Alagoas | 1087 | 43 | 77 | 65 | 98 |
| Vale São Franciscano da Bahia | Bahia | 900 | 47 | 88 | 66 | 99 |

**^1^** De acordo com o critério utilizado no Tropical Data.

**Tabela 4.** Indicadores relacionados à higiene, saneamento, assistência de saúde e educação em unidades de avaliação selecionadas do Brasil, 2018-19


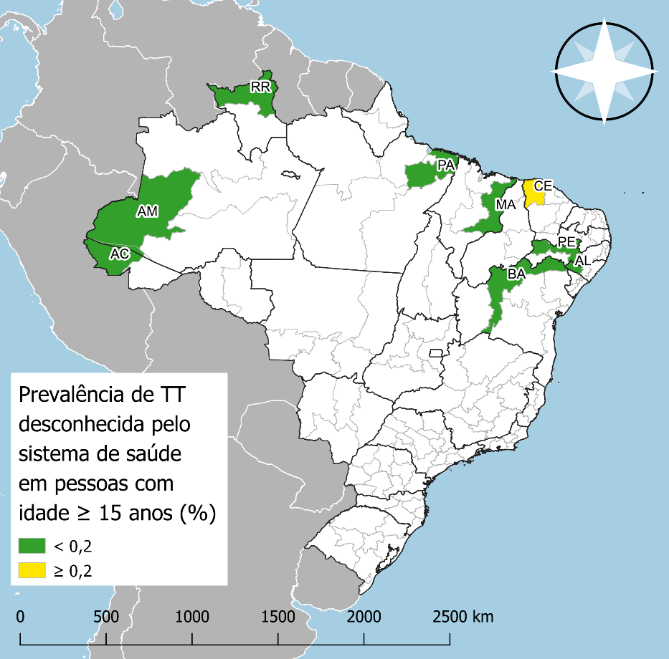
**Figura 1.**

**Legendas das figuras**

**Figura 1.** Prevalência de triquíase tracomatosa (TT) nas Unidades de Avaliação pesquisadas para tracoma. Brasil, 2018–9. AC: Vale do Juruá; AM: Sudoeste Amazonense; RR: Norte de Roraima; PA: Nordeste Paraense; MA: Leste Maranhense; BA: Vale São Franciscano da Bahia; AL: Sertão Alagoano; PE: Sertão Pernambucano; CE: Noroeste Cearense. Os limites e nomes mostrados e as designações usadas neste mapa não implicam expressão de qualquer opinião por parte da Organização Mundial da Saúde e ou a Organização Pan-Americana da Saúde sobre a situação jurídica de qualquer país, território, cidade ou área ou de suas autoridades, ou sobre a delimitação de suas fronteiras ou limites.
